# Oxygenation in cell culture: Critical parameters for reproducibility are routinely not reported

**DOI:** 10.1371/journal.pone.0204269

**Published:** 2018-10-16

**Authors:** Abdullah Al-Ani, Derek Toms, Douglas Kondro, Jarin Thundathil, Yang Yu, Mark Ungrin

**Affiliations:** 1 Biomedical Engineering Graduate Program, University of Calgary, Calgary, AB, Canada; 2 Alberta Diabetes Institute, University of Alberta, Edmonton, AB, Canada; 3 Alberta Children’s Hospital Research Institute, University of Calgary, Calgary, AB, Canada; 4 Department of Comparative Biology and Experimental Medicine, Faculty of Veterinary Medicine, University of Calgary, Calgary, AB, Canada; EFS, FRANCE

## Abstract

Mammalian cell culture is foundational to biomedical research, and the reproducibility of research findings across the sciences is drawing increasing attention. While many components contribute to reproducibility, the reporting of factors that impact oxygen delivery in the general biomedical literature has the potential for both significant impact, and immediate improvement. The relationship between the oxygen consumption rate of cells and the diffusive delivery of oxygen through the overlying medium layer means parameters such as medium depth and cell type can cause significant differences in oxygenation for cultures nominally maintained under the same conditions. While oxygenation levels are widely understood to significantly impact the phenotype of cultured cells in the abstract, in practise the importance of the above parameters does not appear to be well recognized in the non-specialist research community. On analyzing two hundred articles from high-impact journals we find a large majority missing at least one key piece of information necessary to ensure consistency in replication. We propose that explicitly reporting these values should be a requirement for publication.

## Introduction

### Reproducibility

Reproducibility is a critical foundation of science. Shortcomings in this area are a growing concern for preclinical research in particular, given the potential for economic and human health impacts, with some studies reporting more than half of the works they investigated are incompletely reproducible [1–5] (89% [1], 78% [6], 54% [7], and 51% [8]). In the US alone, the economic impact of irreproducibility has been conservatively estimated to exceed US$28 billion annually [9]. Several initiatives have been launched to define and mitigate this problem, including “The Reproducibility Project: Cancer Biology”, which aimed to replicate 50 high impact cancer biology articles [10]. However, this approach has obvious practical and financial limitations, and the flagship project has recently been forced to scale back their objectives from 50 articles to 18 [11]. Perhaps not surprisingly, given the complexity of modern biological research, a major challenge for reproducibility is the publication of research findings with unintended, unrecognized or underreported differences in experimental method [12]. For instance, using mice of different strain, age, or sex can lead to different conclusions, even if other variables are consistent [12–14]. Similarly, passage number, cell identity and culture conditions have been shown to significantly impact reproducibility [15–17]. Other potential causes include poor experimental design, inappropriate statistical analyses [18], and of course outright scientific misconduct [19]. Here, we have chosen to focus on oxygen delivery, as the problem rests on sound and non-controversial theoretical foundations (few researchers would dispute the idea that oxygen impacts cellular phenotype, nor that aqueous solutions provide a significant diffusive barrier to oxygen delivery); and reporting–and awareness–around a few simple parameters can help reduce inconsistencies between experiments without significantly increasing the space allocated to methodology.

### Oxygen levels in culture commonly differ from physiologically-relevant values

In vivo, oxygenation is finely tuned across time and length scales, including via cell-autonomous effects, vasodilation and -constriction, changes in respiratory rate, and vascular remodelling [20,21]. Nearly all of these processes are completely absent in culture, leaving oxygenation levels easily perturbed and dependant on the precise details of culture conditions–which are therefore critical to report to ensure reproducibility.

A common theme in the general literature employing mammalian cell culture is the maintenance of cells in non-physiological oxygen levels, and the use of inadequate terminology to describe these conditions. In particular, culture of cells in incubators in communication with the ambient atmosphere is often referred to as “normoxia”, while cultures in incubators with lower levels are commonly referred to as “hypoxic” [15,22,23]. In turn, “normoxic” incubators are often erroneously assumed to deliver 20.9% oxygen to the cells, without discussion of other parameters (see below), which–if true–would be substantially higher than the normal levels experienced by even well perfused tissues such as the lung parenchyma [24–27]. To avoid ambiguity, we will adopt the use of the recently coined term “physoxia” [26] to describe the oxygen levels a cell would normally encounter in vivo. We refer to higher oxygen levels as hyperoxia, and lower ones as hypoxia (or “near-anoxia” as they approach the limit of the ability of cultured cells to take up oxygen [15,26,28]).

### Variations in oxygenation significantly impact cells

As a key substrate in the bioenergetics of mammalian cells, oxygen availability dictates metabolic efficiency. Aerobic metabolism allows for the theoretical production of up to thirty-six ATP molecules per glucose molecule consumed [29], in contrast to anaerobic glycolysis, which produces two. Thus, oxygenation has the potential for significant direct impacts on cellular metabolism. As cellular metabolism is able to draw oxygen out of solution even at very low concentrations, sufficiently oxygen starved cultures can even achieve near-anoxic status (2.65•10^−7^ mol/l) [30–32], where cellular respiration is expected to vary linearly with oxygen levels under Michaelis-Menten kinetics [30].

However, local oxygen concentration can also impact gene expression and cellular behaviour more subtly [33–36]. Under some conditions, atmospheric oxygen levels can result in a hyperoxic environment for the cultured cells. Decreases in proliferation rate, reduced plating efficiency and progressive decline in metabolic activity have all been reported as consequences of culture in the presence of excess oxygen [26,37], potentially mediated by the generation of reactive oxygen species [30,38,39]. In the case of stem cells, hyperoxia has been reported to promote differentiation, and change responses to growth factors [24]. On the other end of the spectrum, hypoxia can trigger far-reaching signaling cascades via processes such as the unfolded protein response (UPR), mTOR signaling, and hypoxia-inducible factor (HIF)-mediated gene regulation [25,26,40]. This in turn can lead to reduced metabolic rates, temporary cell cycle arrest, promote maintenance of an undifferentiated state, and upregulate production of pro-angiogenic and pro-survival signals [41–43]. It is of particular importance to note that these can be threshold-mediated responses, where a small shift in oxygen concentration can provoke a disproportionate response [40,44,45].

While researchers who work specifically in the area of oxygenation and cellular metabolism are conscious of factors that affect it [15,46], discussions of reproducibility do not generally consider oxygenation [1–8]. However, as discussed above, if cells in one experiment are hyperoxic and in another, hypoxic, due to the relevant variables not being specified, even if all other aspects of an experimental system are adequately described, significant inconsistencies in behaviour would be quite likely. While the problem of inadequate methodological detail in the literature is well recognized, and a great many other factors also contribute to irreproducibility, we wished to quantitively determine the prevalence of this issue as related specifically to oxygenation, and to raise awareness of specific, simple steps that should be taken to avoid it. We hypothesized that a substantial proportion of articles employing mammalian tissue culture as a research tool do not report sufficient methodological detail to ensure reproducibility of the oxygenation conditions to which the cells were exposed, even in top-tier journals.

## Results and discussion

### Key factors affecting oxygen diffusion

Once steady state conditions are achieved following e.g. medium change, diffusive flux of oxygen through the growth medium to the cells being cultured is governed by multiple factors including cellular O_2_ consumption rate, cell density, O_2_ partial pressure, O_2_ solubility, temperature, media diffusion properties and the height of the media column [47–51]. Although for reasons of complexity we do not address it here, note also that the amount of oxygen delivered directly through polystyrene culture surfaces can be significant [32].

According to Fick’s first law, diffusive flux is proportional to the concentration gradient, which in turn is a function of the difference in concentration between the gas-liquid interface and the culture surface, divided by the thickness of the liquid layer. As the cells consume oxygen at a given rate, the local concentration falls, increasing the gradient (and therefore flux) until equilibrium is attained, and the local oxygen concentration stabilizes at the corresponding value. However, there is an upper limit to diffusive delivery of oxygen in a given system–oxygen levels at the culture surface cannot go below zero, at which point the diffusion gradient (and therefore the flux) cannot be further increased without increasing atmospheric oxygen levels, or reducing the thickness of the liquid layer. Beyond this point, cellular metabolism is necessarily limited (either by adaptive biological processes, or simple physics).

**Fig 1:**
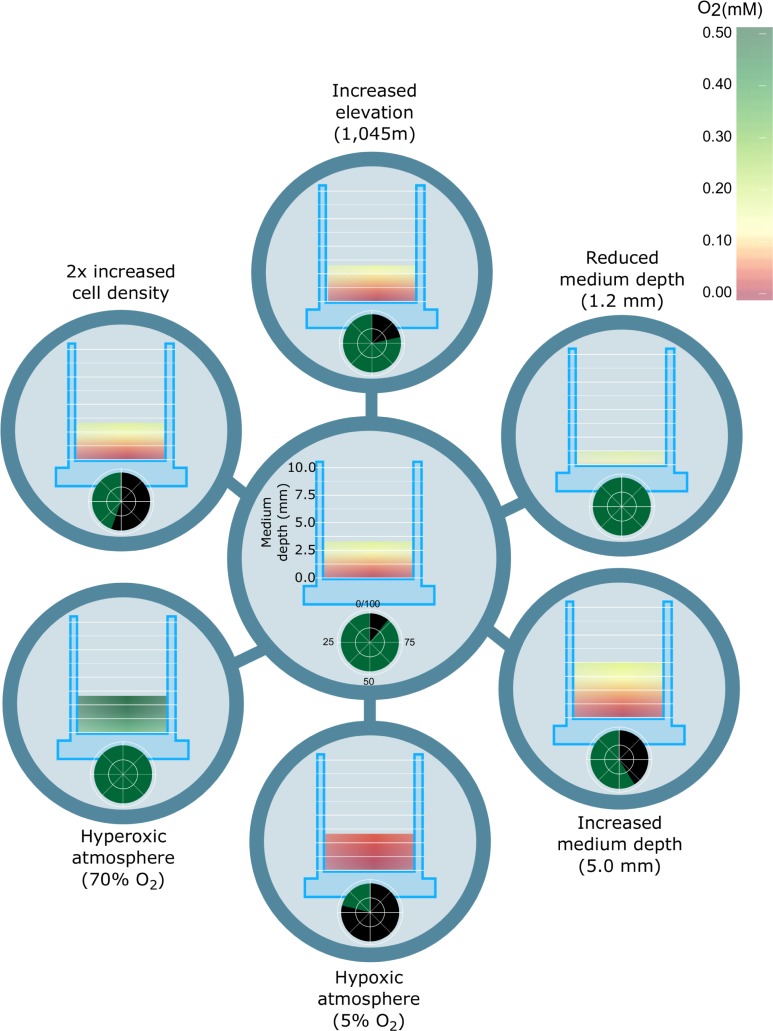
Steady-state oxygen mass transfer in cell culture media. Shown in the centre is a plot of the calculated oxygen concentration (mmol/L) across a media column under culture conditions of $0.3\bar{3}\;cm$ media depth, atmospheric pressure at sea level, a culture density of 200,000 cells / cm^2^, and an oxygen consumption rate for CHO cells of $8.60\times 10^{-17}\frac{mol}{cell∙s}$ [47]. Each condition shown around the perimeter represents the consequences of a change in one variable. At the base of each column the green filling within the circle indicates the fraction of the cells’ metabolic oxygen needs that can be met under each condition.

Modelling this process demonstrates that relatively minor changes to critical variables (Table 1)–within ranges that could realistically occur in routine culture–can result in significant changes to both oxygen delivery per cell and local oxygen concentration around them (**Fig 1**) (for detailed example calculations see **Supplementary Materials**).

### Variables impacting oxygenation are not adequately documented in the literature

**Fig 2:**
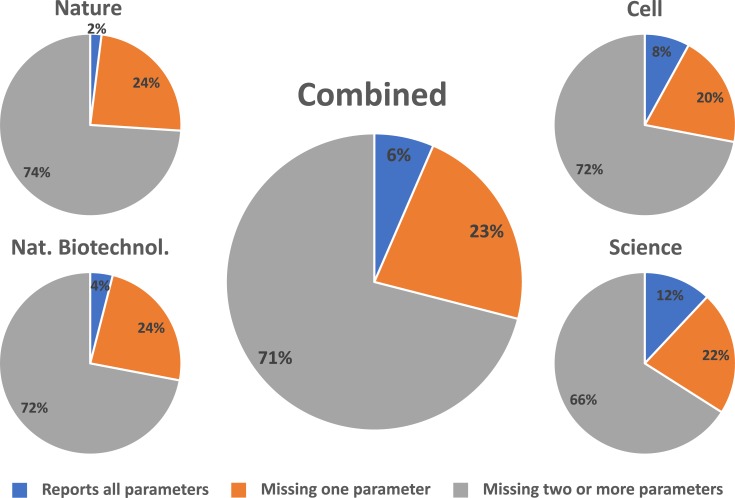
Results from analysis of published work employing mammalian cell culture. Manuscripts were assessed and identified as specifying all of the critical variables (blue), missing only one of the critical variables (orange), or missing multiple critical variables (grey).

While the ideal study would include the actual oxygen levels at the culture surface, key factors to calculate this value such as the specific oxygen consumption rate per cell may not be known, while equipment to directly measure dissolved oxygen is not universally available. Therefore, we focused on parameters which would be minimally required to ensure *reproducibility* of oxygenation conditions, even if the absolute value was not determined (**Table 1**). We assessed fifty recent articles making use of mammalian tissue culture (see Materials and Methods) from each of four widely-recognized high-impact journals: Nature Biotechnology, Nature, Cell and Science. Out of the two hundred papers examined (**Fig 2**), 71% were missing values for more than one critical variable, 23% were missing only one and 6% had all the necessary data. None of the papers attempted to calculate the oxygen levels in the microenvironment around the cells.

**Table 1:** Critical factors determining oxygen delivery to the culture surface [32,47].

FACTOR	DESCRIPTION
**CULTURE VESSEL**	In combination with medium volume, determines medium depth^a^
**CELL TYPE**	Oxygen consumption rates vary widely between cell types^b^
**SEEDING DENSITY**	With cell type, determines oxygen consumption rate per unit area^c^
**MEDIA VOLUME**	With culture vessel geometry, determines medium depth^a^

^a^Medium depth in turn determines the diffusive barrier to oxygen delivery

^b^As there can be significant variation between cells nominally of the same line [30,52–57], cell type and history should be specified as precisely as possible

^c^While theoretically if cell density is reported at time of plating, a replicate experiment will duplicate the density after a fixed period of expansion, ideally cell density at time of harvest / experimentation should also be reported

While this does not necessarily mean that an attempt to reproduce the findings of a given publication would fail–the experimenters might choose conditions that result in a relatively similar oxygen environment, or alterations in gene expression or metabolism due to altered oxygenation might not dramatically affect the specific finding in question–it does draw our attention to a substantial area of risk, and provides a potential explanation that should be considered when unable to recapitulate a published finding.

### Variables impacting oxygenation should be documented in all manuscripts employing mammalian cell culture

The prevalence of the problem demonstrates that a systemic solution is urgently required to ensure reproducibility of published experiments.

We propose that journal editors require all manuscripts employing mammalian cell culture to include in the materials and methods a section entitled “Oxygenation considerations”, which would at a minimum, explicitly include cell type, culture chamber specifications, media volume, and cell density both at time of seeding and of experimentation, unless those values are already included elsewhere, in which case their location should be specified in the cover letter to the editor.

Note that this information is necessary but not sufficient to ensure reproducibility. Many common cell lines are informally transferred between laboratories, potentially accumulating genetic and epigenetic alterations, and in many cases are (deliberately or otherwise) selected on the basis of phenotypes such as clonal expansion capacity. It would not be surprising for metabolism (and therefore oxygen consumption rates) to differ between two cultures of what is nominally the same cell line. Where possible, oxygen consumption rates and/or measured oxygen concentrations should be reported, as should other factors that might influence oxygen delivery, including high levels of vibration or frequent movement of culture vessels (e.g. in a crowded incubator, which would result in mixing of the medium and accelerate delivery of oxygen); obstructions to air flow below the culture vessel (which would reduce oxygen delivery through the culture surface); or the fact that a given laboratory is at a particularly high altitude (which would reduce absolute oxygen concentration in the atmosphere).

In addition to the parameters listed above, oxygen delivery to cultured cells can be influenced by other factors and reporting these would further ensure accurate replication of experiments: cell density at time of experiment, culture temperature, and partial pressure of O_2_ (Table 2). Theoretically, cell density should be consistent given the same starting density, culture conditions, and elapsed time, however explicitly reporting this value would remove a potential source of variability. Culture temperature is often assumed to be 37°C if not explicitly reported, and if not specified the partial pressure of O_2_ is generally considered to reflect exposure to atmospheric oxygen, however it is important to note that this is a function of the altitude of the location at which the culture experiment was carried out, and this value should also be reported.

**Table 2:** Additional parameters that affect the amount of delivered oxygen to cultured cells.

FACTOR	EFFECT	CITATION
**TEMPERATURE**	A) Increases in temperature cause conflicting effects of increasing the diffusion coefficient while decreasing oxygen solubility. In distilled water at 25°C that is heated to 37°C the combined effect increases the flux of oxygen by approximately 15%.	[15,58–60]
**PARTIAL PRESSURE OF OXYGEN**	A) An increase in altitude decreases the equilibrium dissolved oxygen; in our laboratory in Calgary (elev. 1045m) atmospheric pressure (and hence maximum oxygen flux) is about 13% lower than at sea level. Note that meteorological pressure reports for a given location commonly refer to “Altimeter setting” pressure (normalized to altitude), as opposed to the true barometric “Station Pressure”, which can give the false impression that local air pressure is similar to that found at sea level. B) An increase in pressure increases the solubility of oxygen, so doubling the ambient air pressure would double the oxygen flux in a system. C) The introduction of humidity and carbon dioxide effectively dilutes other atmospheric components–for dry air moving to saturation (~6% water vapour) and 5% CO2 reduces the partial pressure of oxygen by 11% (or 8% for an initial atmosphere at 50% relative humidity).	[15,30,61,62]
**CONVECTIVE MIXING**	Vibration, large medium heights and temperature gradients will increase convective mixing which will increase the mass transfer of oxygen.	[59,63,64]
**TISSUE CULTURE MEDIA COMPOSITION**	A) Increases in the ionic strength of culture media of reduce the solubility of oxygen and the diffusion coefficient of oxygen; the combined effect is to reduce oxygen flux by approximately 17% compared to distilled water (*using our values in this paper, see supplementary for the range of values reported in literature).	[15,30,59,65–67]
**HANDLING/ REMOVAL FROM TISSUE CULTURE**	A) Equilibration to a steady-state oxygen profile happens on a time scale of one to several hours, depending on medium depth but also culture chamber details. B) Opening incubator doors changes the gas mixture in the incubator, which can take on the order of an hour to equilibrate. This can cause changes in the oxygen concentration in the cell culture media, particularly during experiments in low-oxygen atmospheres, which in turn can have more extended effects.	[15,68]
**TISSUE CULTURE GEOMETRY**	A) Oxygen diffusion through polystyrene varies between culture vessel geometries, and has been reported to be responsible for up to 30% of oxygen delivered to the tissue culture. B) A meniscus causes variation in media column height, which is particularly relevant in small wells such as in a 96 well plate.	[15,32,69]

Although it would be ideal to measure or calculate and report the amount of oxygen delivered per cell, reporting the recommended essential information should be sufficient to reproduce the amount of oxygen available for cultured cells. Where the necessary information is available, we recommend that investigators calculate the approximate oxygenation conditions within their culture systems as this may inform interpretation of their findings.

### Oxygenation status should be considered when interpreting experiments

Methods of empirically determining oxygen concentration at the cell level in a static culture include use of electrode probes [70], florescent oxygen sensor spots [30], embedded florescent reporters [46] or biological indicators [30]. Unfortunately, these methods are expensive and generally not accessible for routine tissue culture monitoring. While continuous monitoring of true oxygen values would be ideal, in its absence mathematical modelling using a simple formulation of Fick’s law applied to static tissue culture provides an informative, if imperfect alternative [47]. To facilitate use of such a model, we have developed a spreadsheet calculator (**S2 File**) that allows researchers to estimate the theoretical oxygenation status of their cell cultures. The expected concentration of dissolved oxygen in an incubator under atmospheric oxygen (18.6% see [15]) at 37°C ranges from 175 μM –204 μM [30,47,59,71,72]. For our calculations we used a value of 179 μM [73] (the solubility in distilled water is 200 μM [60]. The estimated oxygen diffusion coefficient vary in literature from 0.976–3.00 x10-5 cm^2^/s [28,32,46,47,50,59,67,71,72]. The value used here (2.86 cm^2^/s [32]) is fairly conservative, and lower diffusion coefficients predict even more dramatic oxygen limitations.

Gas mixtures and atmospheric pressure directly influence the equilibrium concentration of oxygen at the air-liquid interface. The depth of the liquid and the consumption rate of oxygen by the cells allows calculation of the theoretical concentration gradient through the media. Depending on the cell density and user-specified oxygen consumption rate (either measured experimentally or obtained from the literature [30] the maximum flux of oxygen through the media may or may not be sufficient to meet the cells’ oxygen stated requirements. If the maximum flux exceeds the total consumption, the calculated culture-surface oxygen concentration at which equilibrium is reached is displayed. Otherwise, the cells will consume oxygen down to near anoxic levels [31], and the proportion of the cells’ nominal oxygen consumption rate that cannot be met under the specified culture conditions is calculated and displayed.

## Conclusion

Varying O_2_ levels clearly have significant potential to impact culture phenotype, and while this fact is widely recognized in the community, it appears not to be taken into account on a routine basis. While any given case of irreproducibility can not necessarily be attributed to a lack of details concerning cell oxygenation status, in order to promote reproducibility in the scientific literature, journal editors should ensure that all published manuscripts employing mammalian tissue culture specify the four critical oxygenation parameters. Experimenters should routinely estimate oxygenation conditions in their culture systems and consider them in their analyses.

## Materials and methods

Paper selection:

The following search term was used to retrieve articles, with “JOURNALNAME” replaced by each of: “Nature”, “Nature Biotechnology”, “Cell” and “Science”:

("JOURNALNAME"[Journal]) AND ((mammalian[Text Word] OR mammal[Text Word] OR human[Text Word] OR mouse[Text Word] OR rat[Text Word] OR rabbit[Text Word] OR hamster[Text Word]) AND (culture[Text Word] OR cultured[Text Word] OR cell culture[Text Word] OR tissue culture[Text Word])) AND ("2000/01/01"[Date—Publication]: "2016/06/01"[Date—Publication])

The resulting articles were then evaluated manually (**S1 Fig**) to restrict them to primary research articles reporting mammalian cell culture sustained for a minimum of twenty-four hours, and the most recent 50 papers from each journal were selected for scoring.

Scoring papers:

Each paper was scored by two evaluators independently, and any discrepancies were subsequently resolved by discussion to establish a consensus score. A conservative list of critical parameters was identified (**Table 1**), whose absence provides a challenge for efforts to reproduce the published work precisely: cell type, media volume, culture chamber specifications, and seeding density. Each paper was scored as including all parameters; missing one parameter; or missing two or more parameters.

## Supporting information

S1 File Includes sample calculations for oxygen delivery under certain example conditions, and paper scoring criteria. (DOCX)

S2 File Oxygen delivery calculator. This spreadsheet makes initial predictions about oxygen delivery to cells cultured under a given set of conditions. As the model is fairly basic, these predictions should not be considered as definitive, but rather as a starting point for deeper consideration of culture behaviour.
(XLSX)

S3 File Raw scores of the evaluated papers. (XLSX)

S1 Fig Summary of paper selection process. A PubMed search was used to retrieve articles from Nature, Nature Biotechnology, Cell and Science with publication date between 2016/06/30–2000/01/01 containing the key words (“Mammalian” OR “Mammal” OR “human” OR “mouse” OR “rat” OR “rabbit” OR “hamster”) AND (“culture” OR “cultured” OR “cell culture” OR “tissue culture”). The resulting articles were then evaluated manually to restrict them to primary research articles reporting mammalian cell culture sustained for a minimum of twenty-four hours, and the most recent 50 papers from each journal were selected for scoring.
(TIF)
