# Supplementary material for: Oxygenation in cell culture: Critical parameters for reproducibility are routinely not reported
Source: PLoS One. 2018 Oct 16;13(10):e0204269. doi: 10.1371/journal.pone.0204269 (PMC6191109; doi:10.1371/journal.pone.0204269)
Supplement: S1 File — (DOCX) [file pone.0204269.s001.docx]

**Sample calculation:**

For the convenience of the reader, a spreadsheet is included in the supplementary materials that implements these calculations for user-specified input conditions.

**
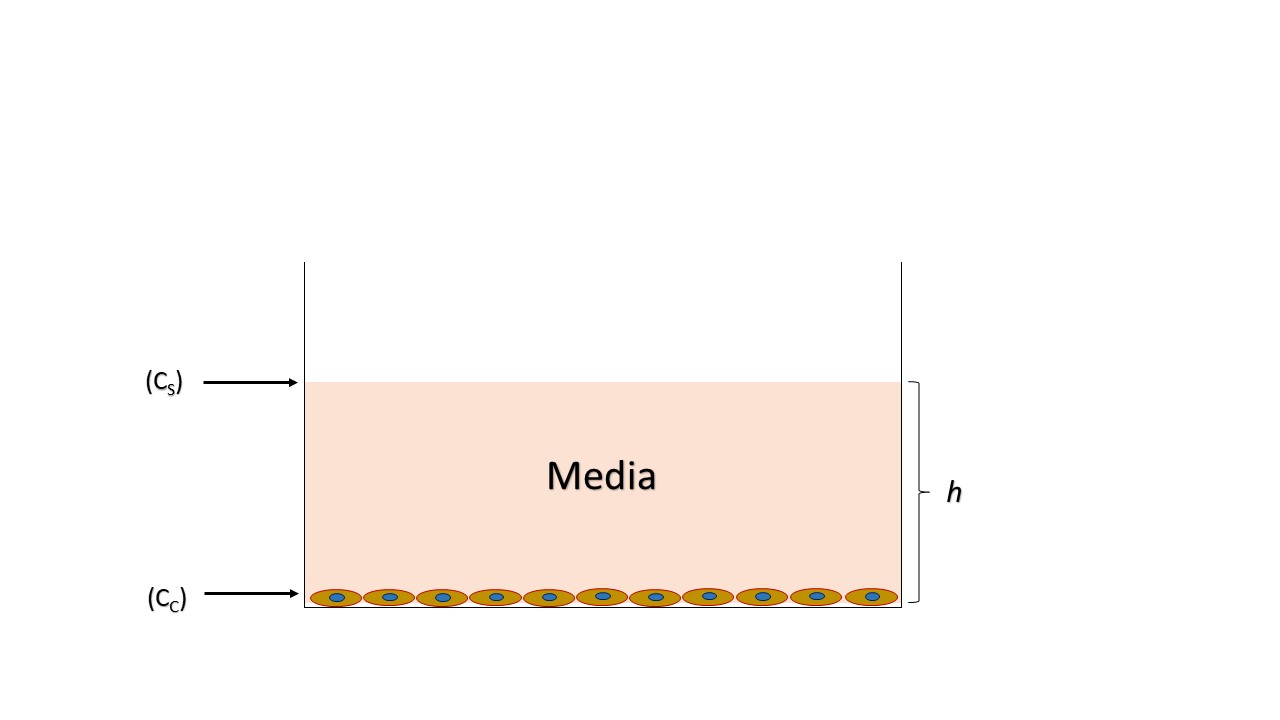
**

Fick’s law ^1^ $J=-D \times\nabla C$

Assuming one-dimensional diffusion at steady state, Fick’s law is simplified^1^ to equation (1):

$J=-D \times\frac{\mathrm{dC}}{dz}$ (1)

By applying our upper and lower boundary conditions where${C= C}_{S}$ at $z=0$and ${C= C}_{C}$ at $z=h$, Fick’s law becomes equation (2)

$$J=-D \times\frac{\Delta C}{\Delta z}$$

$$J=-D \times\frac{C_{S}- C_{c}}{h}$$

 (2)

Where:

J O_2_ flux

$\nabla C$ Gradient of concentration

$\Delta C$ change in concentration

D Diffusion coefficient ^2^ for O_2_ in DMEM media at 37 °C in $\frac{m^{2}}{s}$., D= $2.86\times{10}^{-9}\frac{m^{2}}{s}$ (3)

h liquid height; for this sample calculation

$C_{C}$ [O_2_] at the cells

$C_{S}$ [O_2_] at the air interface

**Assumptions:**

- The equation is solved in one dimension – these calculations ignore meniscus effects at the edge of the culture well^1^
- The tissue culture media is a dilute solution with respect to dissolved O_2_ – which means that we assume that the diffusion constant (D) is in fact constant and doesn’t vary significantly with O_2_ concentration^1^
- These calculations do not take into account convective medium flows that could arise from vibration^2^
- For purposes of this simplified example, oxygen that diffuses through the wall of the container is ignored^2^

**Calculating C_S_ from Henry’s Law:**

$P\cdot\gamma_{O_{2}}= H\cdot x^{*}$ (4)

‘P’ is pressure of the gas mixture above the medium, while ‘$\gamma_{O_{2}}$’ is the mole fraction of O_2_ within this mixture. ‘H’ is Henry’s constant, and ‘$x^{*}$’ is equilibrium mole fraction of O_2_ in the medium immediately adjacent to the interface.

According to the literature,^3,4^ at 37 ^o^C and atmospheric pressure, H is estimated to be 1047 $\frac{L \cdot atm}{mol}$.

Note that under standard culture conditions, the partial pressure of oxygen in the atmosphere must be corrected to reflect the oxygen being displaced by the 5% CO_2_ and 6% water vapor in the incubator.^5^

Therefore (starting with dry air in this example for simplicity’s sake):

$\gamma_{O_{2}}=0.209 \cdot\left( 1.00-0.05-0.06 \right)=18.6\%$

$$P\cdot\gamma_{O_{2}}= H\cdot x^{*}$$

$$\frac{1 atm \cdot18.6\%}{1047 \frac{L \cdot atm}{mol}}= C_{s}$$

$$\boldsymbol{C}_{\boldsymbol{S}}\boldsymbol{=1.79 \times}\boldsymbol{10}^{\boldsymbol{-4}}\frac{\boldsymbol{mol}}{\boldsymbol{L}}$$

We achieve the maximum possible O_2_ flux (J_max_) when the concentration gradient is maximal; this in turn results when oxygen concentration at the cells is minimal. It has been reported^6^ that some cells at least are able to maintain 50% of their normal metabolic activity down to C_C_ = $2.6 \times{10}^{-7} \frac{mol}{L}$. As this value is almost three orders of magnitude smaller than C_S_, we approximate it as zero for the purpose of calculating J_max_, thus:

$\Delta C= C_{S}-C_{C}$ (5)

${\Delta C}_{Max}\cong C_{S}$ (6)

$$\Delta C\cong\boldsymbol{1.79\times}\boldsymbol{10}^{\boldsymbol{-4}}\frac{\boldsymbol{mol}}{\boldsymbol{L}}$$

**Example 1: Here we use a standard 6 well plate from Thermo Fisher Scientific, of area 9 cm^2^, containing 3 mL of medium per well.**^7^ ***–*** *The height of the medium column h is therefore (3 cm^3^ / 9 cm^2^ =* $0.3\bar{3} cm$*)*

$$J_{Max}=-D \times\frac{{\Delta C}_{Max}}{z}$$

$$J_{Max}=2.86\times{10}^{-5}\frac{{cm}^{2}}{s} \times\frac{1.79 \times{10}^{-4}\frac{mol}{L}}{0.3\bar{3} cm}$$

$$J_{Max}=1.53\times{10}^{-8}\frac{cm \cdot mol}{s\cdot L} \times\frac{L}{1000 {cm}^{3}}$$

$$\boldsymbol{J}_{\boldsymbol{Max}}\boldsymbol{=}\boldsymbol{1.53\times}\boldsymbol{10}^{\boldsymbol{-11}}\frac{\boldsymbol{mol}}{\boldsymbol{cm}^{\boldsymbol{2}}\boldsymbol{\cdot s}}$$

We then assess whether J_Max_ (the maximum rate at which oxygen could be delivered to the cells through the overlying medium) is greater or less than the amount of oxygen required by the cells for full metabolism (J_req_).

If J_req_ > J_max_, then there is an oxygen deficit in the system (normal cellular metabolism would consume more oxygen than can be delivered). This deficit may be described by reporting the fraction of normal oxygen demand that remains unmet:

$\frac{\boldsymbol{J}_{\boldsymbol{req}}\boldsymbol{-}\boldsymbol{J}_{\boldsymbol{Max}}}{\boldsymbol{J}_{\boldsymbol{req}}}$ **(7)**

Alternatively, since J is proportional to $\Delta C$ (see equation 2 above), if J_req_ ≤ J_Max_ then at equilibrium the net flow J is less than the maximum possible flow J_Max_, and we can calculate the $\Delta C$ (and thereby C_C_) necessary to drive that flow J by setting J = J_req_. Thus:

$\frac{\boldsymbol{J}_{\boldsymbol{req}}}{\boldsymbol{J}_{\boldsymbol{Max}}}\boldsymbol{=}\frac{\boldsymbol{\Delta C}}{\boldsymbol{\Delta C}_{\boldsymbol{Max}}}$ **(8)**

$$\boldsymbol{\Delta C=}\frac{\boldsymbol{\Delta C}_{\boldsymbol{Max}}\boldsymbol{\cdot}\boldsymbol{J}_{\boldsymbol{req}}}{\boldsymbol{J}_{\boldsymbol{Max}}}$$

$\boldsymbol{C}_{\boldsymbol{C}}\boldsymbol{=}\boldsymbol{C}_{\boldsymbol{S}}\boldsymbol{- \Delta}$**C (9)**

**For instance, if we culture Chinese hamster ovary (CHO) cells in our example system:**

We start with a reported OCR value for CHO cells of $8.60 \times{10}^{-17}\frac{mol}{cells \cdot s}$ from the literature.^8^

CHO cell lines have been reported to achieve between $2 \times{10}^{5}-4 \times{10}^{5} cells/\mathrm{cm}^{2}$ cell density at confluency in standard cell culture surfaces.^9^ Using the lower end of this range for our sample calculation:

$$\boldsymbol{J}_{\boldsymbol{req}}= \frac{8.60 \times{10}^{-17}mol}{cell x s}\times\frac{2 \times{10}^{5} cell}{{1 cm}^{2}}$$

$$\boldsymbol{J}_{\boldsymbol{req}}\boldsymbol{=}\frac{\boldsymbol{1.72\times}\boldsymbol{10}^{\boldsymbol{-11}}\boldsymbol{mol}\boldsymbol{O}_{\boldsymbol{2}}}{\boldsymbol{cm}^{\boldsymbol{2}}\boldsymbol{\cdot s}}$$

Note that even though we calculated using the lower end of the reported cell density range, this is greater than the amount of oxygen that could be delivered through the medium column.

**Since** $\boldsymbol{J}_{\boldsymbol{req}}\boldsymbol{>}\boldsymbol{J}_{\boldsymbol{Max}}\boldsymbol{, then oxygen deficit}\left( \boldsymbol{O}_{\boldsymbol{2 def}} \right)\boldsymbol{=}\frac{\boldsymbol{J}_{\boldsymbol{req}}\boldsymbol{-}\boldsymbol{J}_{\boldsymbol{Max}}}{\boldsymbol{J}_{\boldsymbol{req}}}$

$Percentage O_{2 def}=\frac{J_{req}- J_{Max}}{J_{req}} \times100$ (10)

$$Percentage O_{2 def}=\frac{1.72\times{10}^{-11}\frac{mol}{{cm}^{2}\cdot s}- 1.53\times{10}^{-11}\frac{mol}{{cm}^{2}\cdot s}}{1.72\times{10}^{-11}\frac{mol}{{cm}^{2}\cdot s}} \times100$$

$$\boldsymbol{Percentage}\boldsymbol{O}_{\boldsymbol{2 def}}\boldsymbol{=11 \%}$$

Thus, despite being cultured under what would typically be reported as 21% O_2_, these cells are experiencing extremely low local levels of oxygen, and are metabolically limited to about 90% of their normal oxygen consumption. Note however that this simplified calculation does not include oxygen diffusing directly through the tissue culture plasticware^2^

**Example 2: Maintaining the same conditions as example 1, but in this example we use a standard T-25 flask from Thermo Fisher Scientific**^7^ **with 3 mL of medium *–*** *The height of the medium column h is therefore (3 cm^3^ / 25 cm^2^)= 0.12 cm*

$$J_{Max}=-D \times\frac{\Delta C}{z}$$

$$J_{Max}=2.86\times{10}^{-5}\frac{{cm}^{2}}{s} \times\frac{1.79\times{10}^{-4}\frac{mol}{L}}{0.12 cm}$$

$$J_{Max}=4.26\times{10}^{-8}\frac{cm \cdot mol}{s\cdot L} \times\frac{L}{1000 {cm}^{3}}$$

$$\boldsymbol{J}_{\boldsymbol{Max}}\boldsymbol{=}\boldsymbol{4.26\times}\boldsymbol{10}^{\boldsymbol{-11}}\frac{\boldsymbol{mol}}{\boldsymbol{cm}^{\boldsymbol{2}}\boldsymbol{\cdot s}}$$

And $J_{req}= 1.72\times{10}^{-11}\frac{mol O_{2}}{{cm}^{2}\cdot s}$ as previously calculated for Chinese hamster ovary (CHO) cells

**Now** $\boldsymbol{J}_{\boldsymbol{req}}\boldsymbol{<}\boldsymbol{J}_{\boldsymbol{Max ,}}$ **therefore:**

$$\boldsymbol{J=}\boldsymbol{J}_{\boldsymbol{req}}$$

$$\frac{\boldsymbol{J}_{\boldsymbol{req}}}{\boldsymbol{J}_{\boldsymbol{Max}}}\boldsymbol{=}\frac{\boldsymbol{\Delta C}}{\boldsymbol{\Delta C}_{\boldsymbol{Max}}}$$

$$\boldsymbol{\Delta C=}\frac{\boldsymbol{\Delta C}_{\boldsymbol{Max}}\boldsymbol{\cdot}\boldsymbol{J}_{\boldsymbol{req}}}{\boldsymbol{J}_{\boldsymbol{Max}}}$$

$$\boldsymbol{\Delta C=}\frac{1.79 \times{10}^{-4}\frac{mol}{L} \cdot\frac{1.72\times{10}^{-11} mol O_{2}}{{cm}^{2}\cdot s}}{4.26\times{10}^{-11}\frac{mol}{{cm}^{2}\cdot s}}$$

$$\boldsymbol{\Delta C= 7.22} \times{10}^{-5}\frac{mol}{L}$$

$$\boldsymbol{C}_{\boldsymbol{C}}\boldsymbol{=}\boldsymbol{C}_{\boldsymbol{S}}\boldsymbol{- \Delta C}$$

$$C_{Cells}=1.79 \times{10}^{-4}\frac{mol}{L} - 7.22\times{10}^{-5}\frac{mol}{L}$$

$\boldsymbol{C}_{\boldsymbol{Cells}}\boldsymbol{=}\boldsymbol{1.06 \times}\boldsymbol{10}^{\boldsymbol{-4}}\frac{\boldsymbol{mol}}{\boldsymbol{L}}$, which is equivalent to oxygen concentration in a medium in equilibrium with air at 12.6% O_2_ at sea level (Henry’s law calculation not shown)

**Example 3: Maintaining the same conditions as example 1, but in this example we use a 24 well plate (area 2 cm^2^ per well) from Thermo Fisher Scientific**^7^ **containing 1 mL of media per well *–*** *The height of the medium column h is therefore (1 cm^3^ / 2 cm^2^) = 0.5 cm*

$$J_{Max}=-D \times\frac{{\Delta C}_{Max}}{z}$$

$$J_{Max}=2.86\times{10}^{-5}\frac{{cm}^{2}}{s} \times\frac{1.79 \times{10}^{-4}\frac{mol}{L}}{0.5 cm}$$

$$J_{Max}=1.02\times{10}^{-8}\frac{cm \cdot mol}{s\cdot L} \times\frac{L}{1000 {cm}^{3}}$$

$$\boldsymbol{J}_{\boldsymbol{Max}}\boldsymbol{=}\boldsymbol{1.02\times}\boldsymbol{10}^{\boldsymbol{-11}}\frac{\boldsymbol{mol}}{\boldsymbol{cm}^{\boldsymbol{2}}\boldsymbol{\cdot s}}$$

**From the previous examples,** $\boldsymbol{J}_{\boldsymbol{req}}\boldsymbol{=}\frac{\boldsymbol{1.72\times}\boldsymbol{10}^{\boldsymbol{-11}}\boldsymbol{mol}\boldsymbol{O}_{\boldsymbol{2}}}{\boldsymbol{cm}^{\boldsymbol{2}}\boldsymbol{\cdot s}}$

**Since** $\boldsymbol{J}_{\boldsymbol{req}}\boldsymbol{>}\boldsymbol{J}_{\boldsymbol{Max}}\boldsymbol{, then oxygen deficit}\left( \boldsymbol{O}_{\boldsymbol{2 def}} \right)\boldsymbol{=}\frac{\boldsymbol{J}_{\boldsymbol{req}}\boldsymbol{-}\boldsymbol{J}_{\boldsymbol{Max}}}{\boldsymbol{J}_{\boldsymbol{req}}}$

$$Percentage O_{2 def}=\frac{J_{req}- J_{Max}}{J_{req}} \times100$$

$$Percentage O_{2 def}=\frac{\frac{1.72\times{10}^{-11} mol}{{cm}^{2}\cdot s}- 1.02\times{10}^{-11}\frac{mol}{{cm}^{2}\cdot s}}{\frac{1.72\times{10}^{-11} mol}{{cm}^{2}\cdot s}} \times100$$

$$\boldsymbol{Percentage}\boldsymbol{O}_{\boldsymbol{2 def}}\boldsymbol{=41 \%}$$

In this case, the cells would be experiencing a substantial oxygen deficit.

**Note that in the preceding three examples, the same cells, cultured at the same density, in different culture vessels under commonly used volumes of growth medium, are expected to experience dramatically different oxygen environments, ranging from physiologically hyperoxic, to oxygen-limited near-anoxic.**

**Scoring Papers:**

Each paper was scored by two evaluators independently, and any discrepancies were subsequently resolved by discussion to establish a consensus score. A conservative list of critical parameters was identified, whose absence provides a challenge for efforts to reproduce the published work precisely: cell type, media volume, culture chamber specifications, and seeding density. Each paper was scored as including all parameters; missing one parameter; or missing two or more parameters.

In addition to the parameters listed above, oxygen delivery to cultured cells can be influenced by other factors and reporting them would further ensure reproducible oxygen delivery. Although these factors did not influence our current conservative scoring criteria, our evaluators checked if they were reported in each paper. These parameters include: cell number at confluence, culture temperature, and partial pressure of O_2_. Culture temperature is often assumed to be 37 °C if not explicitly reported, and if not specified the partial pressure O_2_ is generally considered to reflect exposure to atmospheric oxygen, however it is important to note that this is a function of the altitude of the location at which the culture experiment was carried out, and this value should also be reported.

Ideally authors would also calculate or measure the Oxygen Consumption Rate (OCR) of the culture and O_2_ flux, however for technical reasons this is often not practical.

**References**

1. Cussler, E. L. *Diffusion: mass transfer in fluid systems*. (Cambridge university press, 2009).

2. Randers-Eichhorn, L., Bartlett, R. A., Frey, D. D. & Rao, G. Noninvasive oxygen measurements and mass transfer considerations in tissue culture flasks. *Biotechnol. Bioeng.* **51,** 466–478 (1996).

3. Vendruscolo, F., Rossi, M. J., Schmidell, W. & Ninow, J. L. Determination of Oxygen Solubility in Liquid Media. *ISRN Chem. Eng.* **2012,** 1–5 (2012).

4. Rettich, T. R., Battino, R. & Wilhelm, E. Solubility of gases in liquids. 22. High-precision determination of Henry’s law constants of oxygen in liquid water fromT=274 K toT=328 K. *J. Chem. Thermodyn.* **32,** 1145–1156 (2000).

5. Dean, J. A. Lange’s handbook of chemistry. *Mater. Manuf. Process.* **5,** 687–688 (1990).

6. Froese, G. The respiration of ascites tumour cells at low oxygen concentrations. *Biochim Biophys Acta* **57,** 509–519 (1962).

7. Scientific, T. F. Useful Numbers for Cell Culture: Useful Information for Various Sizes of Cell Culture Dishes and Flasks. *Thermo Fisher Scientific* (2017). Available at: https://www.thermofisher.com/ca/en/home/references/gibco-cell-culture-basics/cell-culture-protocols/cell-culture-useful-numbers.html.

8. Wagner, B. A., Venkataraman, S. & Buettner, G. R. The rate of oxygen utilization by cells. *Free Radic. Biol. Med.* **51,** 700–12 (2011).

9. Camire, J. & Upton, T. Adherent Growth of CHO cells in Thermo Scienti c HyClone SFM4CHO-A medium using Corning ® CellBIND ® Surface Culture Flasks. *Thermo Scientific* 5–6 (2007).
