# Supplementary figures and images for: Oxygenation in cell culture: Critical parameters for reproducibility are routinely not reported
Source: PLoS One. 2018 Oct 16;13(10):e0204269. doi: 10.1371/journal.pone.0204269 (PMC6191109; doi:10.1371/journal.pone.0204269)

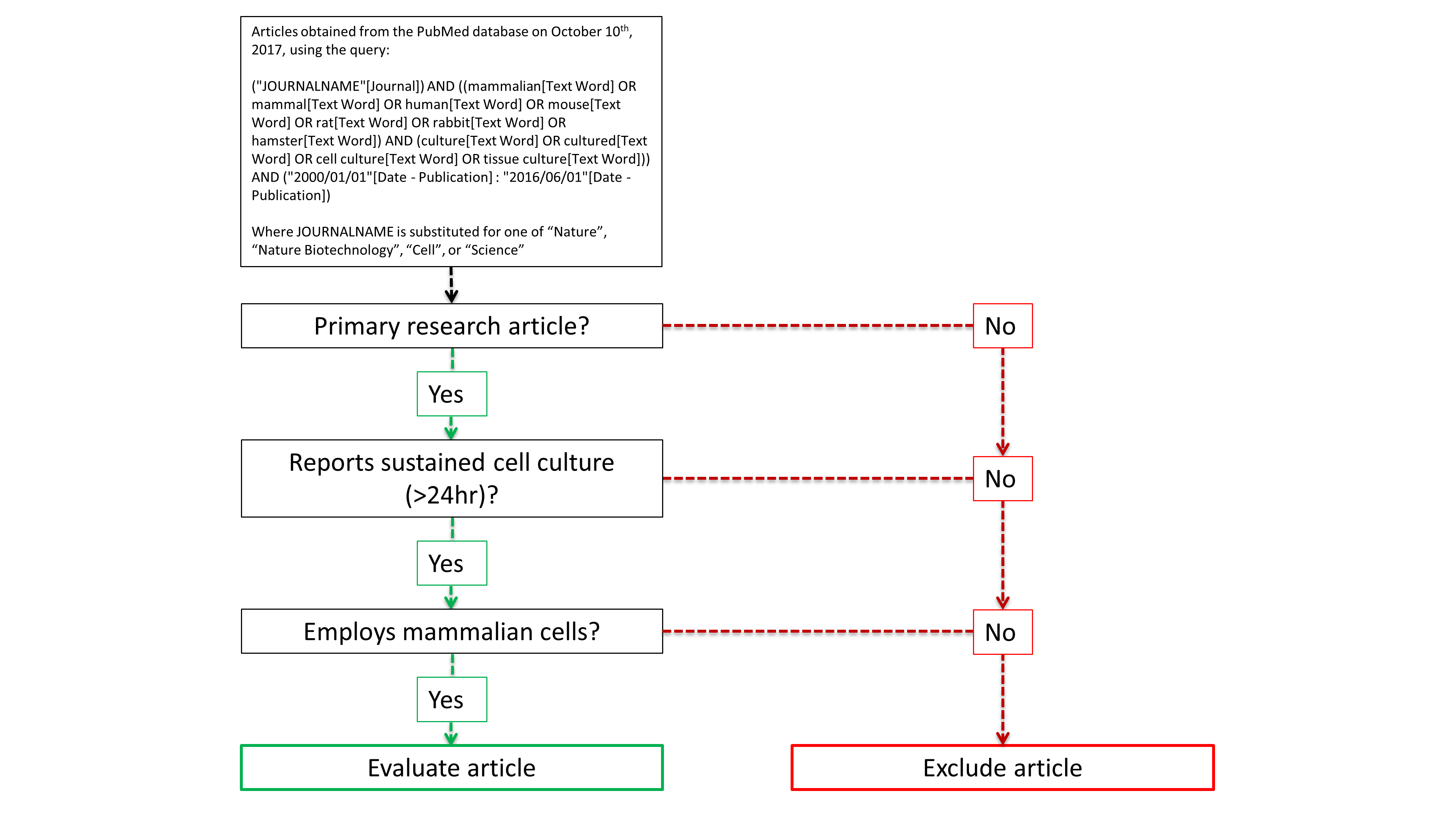

Supplement: S1 Fig — A PubMed search was used to retrieve articles from Nature, Nature Biotechnology, Cell and Science with publication date between 2016/06/30–2000/01/01 containing the key words (“Mammalian” OR “Mammal” OR “human” OR “mouse” OR “rat” OR “rabbit” OR “hamster”) AND (“culture” OR “cultured” OR “cell culture” OR “tissue culture”). The resulting articles were then evaluated manually to restrict them to primary research articles reporting mammalian cell culture sustained for a minimum of twenty-four hours, and the most recent 50 papers from each journal were selected for scoring. (TIF) [file pone.0204269.s004.tif]
